# ECM Characterization Reveals a Massive Activation of Acute Phase Response during FSGS

**DOI:** 10.3390/ijms21062095

**Published:** 2020-03-18

**Authors:** Eva Nora Bukosza, Christoph Kornauth, Karin Hummel, Helga Schachner, Nicole Huttary, Sigurd Krieger, Katharina Nöbauer, André Oszwald, Ebrahim Razzazi Fazeli, Klaus Kratochwill, Christoph Aufricht, Gabor Szénási, Peter Hamar, Christoph A. Gebeshuber

**Affiliations:** 1 Institute of Translational Medicine, Semmelweis University Budapest, Tűzoltó u 37-47, 1094 Budapest, Hungary; nora.bukosza@gmail.com (E.N.B.); szenasi.gabor@med.semmelweis-univ.hu (G.S.); hamar.peter@med.semmelweis-univ.hu (P.H.); 2 Clinical Institute for Pathology, Medical University Vienna, Währinger Gürtel 18-20, 1090 Vienna, Austria; Christoph.Kornauth@meduniwien.ac.at (C.K.); helga.schachner@meduniwien.ac.at (H.S.); nicole.huttary@meduniwien.ac.at (N.H.); sigurd.krieger@meduniwien.ac.at (S.K.); andre.oszwald@meduniwien.ac.at (A.O.); 3 Clinical Division of Hematology and Hemostaseology, Department of Internal Medicine I, Medical University Vienna, Währinger Gürtel 18-20, 1090 Vienna, Austria; 4 Vetcore Facility for Research, University of Veterinary Medicine Vienna, Veterinärplatz 1, 1210 Vienna, Austria; karin.hummel@vetmeduni.ac.at (K.H.); katharina.noebauer@vetmeduni.ac.at (K.N.); ebrahim.razzazi@vetmeduni.ac.at (E.R.F.); 5 Christian Doppler Laboratory for Molecular Stress Research in Peritoneal Dialysis, Department of Pediatrics and Adolescent Medicine, Medical University of Vienna, 1210 Vienna, Austria; klaus.kratochwill@meduniwien.ac.at; 6 Division of Pediatric Nephrology and Gastroenterology, Department of Pediatrics and Adolescent Medicine, Medical University of Vienna, 1210 Vienna, Austria; christoph.aufricht@meduniwien.ac.at

**Keywords:** FSGS, ECM, sclerosis, acute phase response, fibrinogen, complement system

## Abstract

The glomerular basement membrane (GBM) and extra-cellular matrix (ECM) are essential to maintain a functional interaction between the glomerular podocytes and the fenestrated endothelial cells in the formation of the slit diaphragm for the filtration of blood. Dysregulation of ECM homeostasis can cause Focal segmental glomerulosclerosis (FSGS). Despite this central role, alterations in ECM composition during FSGS have not been analyzed in detail yet. Here, we characterized the ECM proteome changes in miR-193a-overexpressing mice, which suffer from FSGS due to suppression of Wilms’ tumor 1 (WT1). By mass spectrometry we identified a massive activation of the acute phase response, especially the complement and fibrinogen pathways. Several protease inhibitors (ITIH1, SERPINA1, SERPINA3) were also strongly increased. Complementary analysis of RNA expression data from both miR-193a mice and human FSGS patients identified additional candidate genes also mainly involved in the acute phase response. In total, we identified more than 60 dysregulated, ECM-associated genes with potential relevance for FSGS progression. Our comprehensive analysis of a murine FSGS model and translational comparison with human data offers novel targets for FSGS therapy.

## 1. Introduction

The kidney glomerulus is essential for the filtration of blood. A sieve-like structure is formed by interdigitating foot processes of podocytes and underlying fenestrated endothelial cells. Those cellular components are separated by the glomerular basement membrane/extra-cellular matrix (GBM/ECM). The glomerular ECM provides a supporting scaffold for podocytes and endothelial cells and integrates signals between cells and their environment, including survival and growth factor signaling [1,2,3]. Dysregulation of ECM deposition and formation of excessive connective tissue histologically manifests as sclerosis and can cause organ dysfunction or failure [4], especially in glomerular diseases [5,6,7].

Focal segmental glomerulosclerosis (FSGS) refers to a histo-pathological pattern of injury characterized by sclerosis in some areas of some glomeruli [8,9]. Heterogeneous underlying causes (e.g., gene mutations, circulating factors, miRNAs, hypertension, drugs, viruses) invariably lead to the common ultrastructural denominator—podocyte foot process effacement—or podocyte loss, which clinically manifests as nephrotic range proteinuria. Some patients respond to corticosteroids or calcineurin inhibitors, but specific treatment is lacking [10]. FSGS frequently progresses to a common diagnostic endpoint defined by pronounced sclerotic lesions causing loss of glomerular function and end-stage renal disease (ESRD) [8,9]. Therefore, the elucidation of changes in the ECM composition during FSGS progression might be instrumental to gain a better understanding of these pathological processes and aid development of novel therapies.

Recently, the composition of the glomerular ECM of humans and mice have been established [11,12,13]. Furthermore, comparison of the glomerular ECM of mice with different genetic backgrounds and basic levels of proteinuria revealed changes in certain proteins, which might enhance disease susceptibility [7]. To gain deeper insight into the changes during FSGS development and progression, we characterized the ECM proteome during FSGS in the murine miR-193a transgenic model by mass spectrometry (MS) analysis. miR-193a-overexpression suppresses Wilms’ tumor 1 (WT1), a transcription factor and master regulator of podocyte identity. Subsequently, this leads to the loss of many WT1 targets essential for podocyte differentiation and homeostasis, which ultimately causes FSGS and kidney failure [14]. We also found significantly enhanced levels of miR-193a in patients suffering from idiopathic FSGS [14]. We complemented our MS data with a candidate search of the RNA level in a murine FSGS model and human FSGS patient data.

## 2. Results

**Figure 1:**
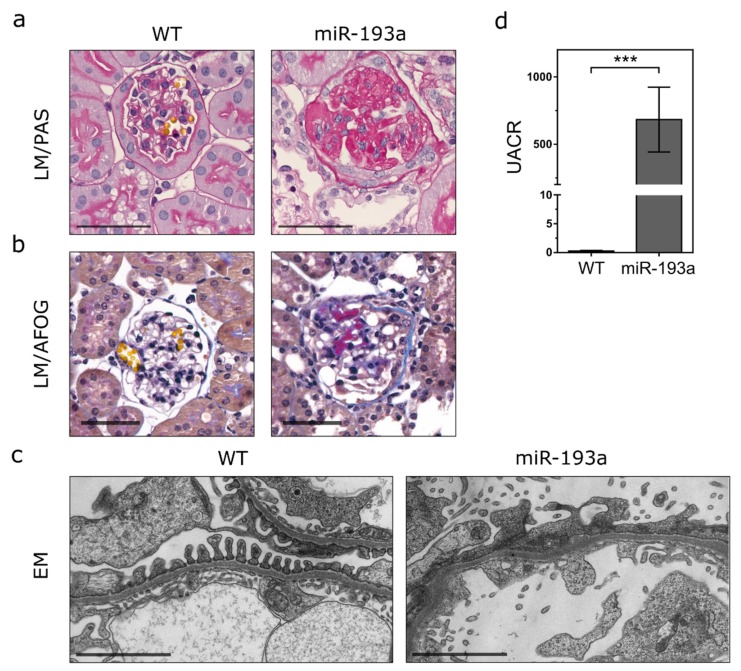
Kidney histology and albuminuria in miR-193a-driven focal segmental glomerulosclerosis (FSGS). Representative images of (**a**) Periodic-acid Schiff (PAS) and (**b**) trichromic Acid Fuchsin Orange G (AFOG) stainings of formalin-fixed paraffin-embedded (FFPE) sections show FSGS, intracapillary cast formation, and extracellular protein deposition in miR-193a mice 8 weeks post induction. (**c**) Electron-microscopy (EM) reveals podocyte foot process effacement, fusion, and detachment in miR-193a FSGS mice 8 weeks post induction of FSGS, but not wild-type control (WT). (**d**) Urinary albumin-to-creatinine ratio (UACR) in wild-type and miR-193a FSGS mice 8 weeks post induction is indicated in g/g. Light microscopy (LM) images scale bar = 100 µm; electron microscopy (EM) images scale bar = 2 µm; values represent means ± SD, *n* ≥ 5/group; unpaired two-tailed Student’s t-test ***: *p* < 0.0001.

By adapting a protocol established by Rachel Lennon and coworkers [7,11], we isolated the ECM of healthy control mice and mice over-expressing miR-193a for 8 weeks [14] (Figure 1a–d). The ECM of four mice per group was characterized by MS analysis.

HPLC–MS analysis identified 1593 proteins with at least two peptides over all samples (Table S1). Manual curation revealed 111 bona fide ECM-associated proteins according to the matrisome resource database [15] plus additional proteins not primarily associated with ECM (e.g., members of the complement pathway, see below) (Table S2). Our findings are in line with a comparable study identifying 115 ECM-associated proteins in two different mouse backgrounds [7]. We found strong and significant expression changes for 18 up- and two downregulated proteins in ECM isolates in miR-193a FSGS mice, four proteins were even exclusively detected in FSGS (Table 1.)

**Figure 2:**
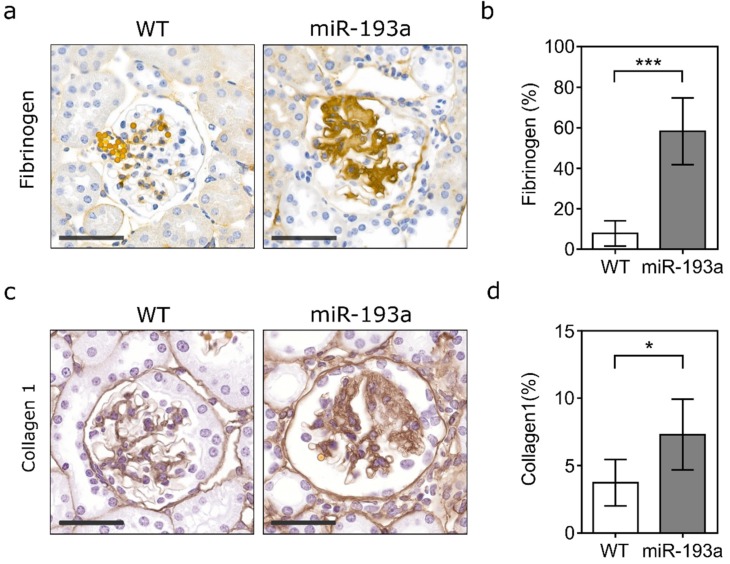
Fibrinogen and collagen 1 in a kidney glomerular matrix. (**a**) Representative images of FFPE sections stained for fibrinogen. (**b**) Fibrinogen-positive area in % of total glomerular tuft area in wild-type control (WT) versus FSGS mice 8 weeks post miR-193a induction. (**c**) Representative images of FFPE sections stained for collagen 1. (**d**) Collagen 1-positive area in % of total glomerular tuft area in wild-type control (WT) versus FSGS mice 8 weeks post miR-193a induction. For PAS and AFOG stainings of the respective sections, see Figure 1. Scale bar = 50 µm; data represent means ± SD, *n* ≥ 4 samples/group, and each data point represents the mean of min. 10 glomeruli/sample; unpaired two-tailed Student’s t-test *: *p* < 0.05; ***: *p* < 0.001.

Especially the activation of the acute phase response in the miR-193a FSGS samples was evident: The complement components C1R and C4B, properdin (CFP), and the complement activator C-reactive protein (CRP) showed a massive increase in protein abundance while the complement membrane attack complex inhibitor CD59A was strongly reduced (Table 1). Furthermore, the fibrinogen α-, β-, and γ-chains (FGA, FGB, FGG) were strongly upregulated during FSGS, although FGA failed to reach significance (Table 1, Table S1, Figure 2a,b). α-globin (HBA1), β1-globin (HBBB1), and β2-globin (HBB-B2) were strongly enhanced, while the polymeric immunoglobulin receptor (PIGR) and the pulmonary surfactant-associated protein D (SFTPD) were even exclusively detected in the FSGS samples.

Our study also revealed a strong increase in several plasma protease inhibitors (ITIH1, SERPINA1, SERPINA3) (Table 1). Protease inhibitors interfere with the turnover and degradation process of ECM material, thereby promoting sclerosis. In line with this, the sclerosis marker Col1a1 was strongly upregulated in miR-193a-driven FSGS (Table 1, Figure 2c,d).

Upregulation was also observed for uromodulin (UMOD; Tamm–Horsfall protein), a master regulator of the immune system and cytokine production in the kidney [16] (Table 1).

In addition, Apolipoprotein A1 (APOA1) (Table 1), the apoprotein of the major high-density lipoprotein (HDL) in plasma was also strongly enhanced.

**Figure 3:**
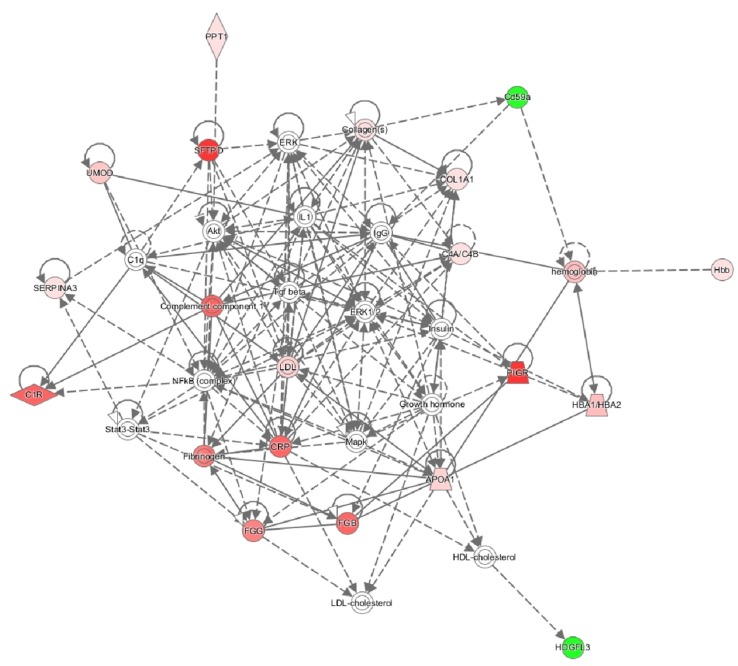
Network of the most strongly dysregulated proteins of the glomerular ECM in FSGS. The dysregulated genes from Table 1 and their interaction were analyzed by Ingenuity Pathway Analysis (red: downregulated; green: upregulated; stronger color means stronger regulation).

Hepatoma-Derived Growth Factor-Related Protein 3 (HDGFL3, HRP3) was the strongest down-regulated protein. HDGFL3 can be secreted and exerts mitogenic functions, especially on endothelial cells [17] (Table 1). Some of the GBM and core structural ECM proteins were also increased (including COL12A1, COL3A1, and TINAG), although significance was not reached (Table S1). Ingenuity pathway analysis confirmed activation of acute phase response as the most significant alteration (Figure 3).

While MS-based analysis of the ECM identifies proteins independent of their origin—in our case several liver products were identified—it bears the risk of missing proteins with low abundance, weak ECM interaction, or generally unsuitable for MS analysis. To overcome the possible loss of such proteins from our analysis, in a second approach, we aimed to identify additional candidate ECM genes with relevance for FSGS. We overlapped genes > 3-fold dysregulated in both miR-193a-overexpressing glomeruli five weeks post FSGS induction as published before by us [14] and of glomeruli of human FSGS patients published by the Potter group [18]. We verified the ECM-association of this overlap with the matrisome database [15] or by PubMed searches. This analysis suggested 32 up- and 5 downregulated ECM-associated genes with potential relevance for FSGS on the mRNA level (Table 2). In line with the MS data, several members of the complement cascade (CFI, C2/CFB, C7), UMOD, and SERPINA1 were also strongly upregulated on the RNA level, in both human and mouse FSGS (Table 2). Among the strongest upregulated genes on the RNA level were insulin growth factor binding protein 1 (IGFBP1), angiotensin converting enzyme 2 (ACE2), tubulointerstitial nephritis antigen (TINAG), and osteopontin (OPN, SPP1). The strongest downregulated gene was sclerostin (SOST), an inhibitor of bone morphogenic protein (BMP) signaling (Table 2). The podocyte survival factor vascular endothelial cell growth factor-alpha (VEGFA) was also lost (Table 2). Among the dysregulated genes were several involved in the regulation of the immune response and the coagulation cascade and genes associated with different cell signaling pathways (Wnt, TGF-β, IGF, VEGF) (Table 2).

## 3. Discussion

In this study, we tried to identify changes in the ECM during miR-193a-driven FSGS. We found a strong acute phase response including upregulation of the complement components C1, C4B, CFP, and CRP. It has been shown before that the complement system promotes inflammation and contributes to FSGS progression and is activated in patients with FSGS [19,20] and that mesangial deposition of C4D is associated with poor renal survival in patients with primary FSGS [21]. This hyperactive complement cascade might also be responsible for the observed strong upregulation of α-globin (HBA1), β1-globin (HBB-B1), and β2-globin (HBB-B2) (Table 1) through the lysis of erythrocytes, which in turn can lead to the production of reactive oxygen species (ROS). ROS have been shown to promote FSGS by different mechanisms, including the stimulation of apoptosis and the upregulation of the transient receptor potential cation channel TRPC6 [22]. Uromodulin is a part of the innate immune system and has been shown to bind to complement components and various cytokines [16].

Another pathway usually activated early after injury and interacting with the complement system is the fibrinogen pathway [23,24,25], which was as well upregulated during miR-193a-driven FSGS. Like complement components, fibrinogen is mostly produced in the liver and plays central roles in blood coagulation, the regulation of inflammation, cytokine expression, fibrosis, and apoptosis [26,27,28]. Fibrinogen is able to alter podocyte homeostasis by binding to Toll-like receptor 4 (TLR4), thereby stimulating p38 phosphorylation and NFκB activation [29]. Complement 3 and fibrinogen chains were also the proteins with the strongest increase in abundance in a recently published study about lipopolysaccharide-induced septic acute kidney injury [30], suggesting that the acute phase response is a very common reaction to kidney injury. PIGR, also associated with the innate immune response and involved in transport of Immunoglobulin A (IgA), was exclusively detected in FSGS samples. Interestingly, PIGR is involved in IgA nephritis and can be upregulated by TLR4 [31,32]. Another member of the innate immune response, SFTPD, was also exclusively detected in the ECM of miR-193a mice.

Furthermore, we found upregulation of several proteases (ITIH1, SERPINA1, SERPINA3), which can turnover ECM components and are therefore of central relevance for the sclerotic process. The HDL component APO1 was also increased in miR-193a-driven FSGS. APOA1 can promote cholesterol efflux, which is able to cause podocyte damage [33]. In general, altered lipid biology may harm podocytes via different pathways [33,34]. A high molecular weight form of APOA1 is a candidate for a circulating factor of recurrent FSGS [35,36]. APOA1 is a binding partner of APOL1, also implicated in FSGS [37].

To reveal additional candidates potentially overseen by MS analysis, we complemented our protein data with ECM-associated genes dysregulated on the RNA level in FSGS patient samples and glomeruli of the miR-193a model [14,18]. This revealed two overlapping candidates (UMOD, SERPINA1) and additional 35 ECM-associated FSGS candidate genes. In line with the MS data, several complement components were also upregulated. The strongest increase in the FSGS patient screen was found for osteopontin, a secreted glycophosphoprotein that can regulate many signaling processes highly relevant for the immune system and FSGS, including interleukins, NFκB, TGF-β, and CD44 [38]. High osteopontin levels were also found in Adriamycin nephropathy and FSGS patients [39,40]. The strongly upregulated genes IGFBP1 and IGFBP3 have been suggested as markers for diabetic nephropathy and chronic kidney disease [41,42]. Furthermore, we found several genes associated with the Wnt and TGF-β pathway, both implicated in FSGS as well as ECM biology [43,44,45].

In summary, we analyzed the changes in the ECM composition during miR-193a-driven FSGS by MS analysis enhanced by an analysis of published RNA screens [14,18]. This combinatorial approach identified more than 60 ECM-associated genes dysregulated in FSGS, including several genes shared with other FSGS models and several novel ones, not discussed in the context of FSGS so far. The most apparent alterations were upregulation of the complement and fibrinogen pathway and of several protease inhibitors. In line with this, pharmacological intervention of thrombin-activated fibrinolysis inhibitor (TAFI) has been shown to ameliorate kidney fibrosis [46]. Therapeutic approaches targeting the complement or fibrinogen and coagulation pathway should therefore receive new attention for the treatment of FSGS.

## 4. Materials and Methods

### 4.1. Transgenic Mice

miR-193a mice (in a Balb/c background) and the respective genotyping were described before [14]. Animal experiments were performed according to approvals from the Austrian Federal Ministry of Science and Research (66.009/0053-II/3b/2014).

### 4.2. Isolation of Mouse Glomeruli and ECM

Isolation was performed based on the magnetic bead method [47] with minor modifications. In brief, anaesthetized mice undertook a midline thoraco-abdominal incision; two ligations were placed, the first one closing the abdominal aorta together with the inferior cava vein below the branching point of the renal vessels, the other one closing the coeliac trunk together with the superior mesenteric artery. Both kidneys were perfused with 100 µL (= 4 × 10^7^) Dynabeads M-450 Tosylactivated (Invitrogen/Life Technologies AS, Oslo, Norway) in ice-cold HBSS (Ca^2+^, Mg^2+^) via a polyethylene tubing extension on top of a 24G needle, inserted and secured into the upper abdominal aorta, while volume outflow was ensured by opening the distally ligated vena cava inferior (VCI). ECM extraction was performed according to a protocol established before [7].

### 4.3. Glomerular ECM Sample Preparation for Analysis by Mass Spectrometry

Proteins bound on the magnetic beads after the glomerular/ECM extraction was dissolved in 10 µL 50 mM TRIS (pH 8.0). For in-solution digestion, an additional 10 µL 8M urea were added (total volume 20 µL). Digestion was performed after reduction with DTT for 30 min at 37 °C and alkylation with iodoacetamide (30 min, 25 °C) using Trypsin/LysC (Promega Corporation, Madison, WI, USA). Digestion with LysC was performed at 37 °C for 4 h. To enable the trypsin digestion after 4 h, the urea concentration was reduced with the addition of 140 µL 50mM TRIS (pH 8.0). After an additional 8 h at 37 °C the digest was stopped by the addition of 0.8 µL of concentrated trifluoro acetic acid (TFA). The magnetic beads were removed, and peptides were collected. After the last sample preparation step, a clean-up of the peptides by C18 columns (Millipore Ziptips C18, Pierce Micro-Spin Columns, Pierce Biotechnology/Thermo Fisher Scientific, Rockford, IL, USA), peptides were injected into an HPLC–MS/MS system.

### 4.4. Hybrid Quadrupole–Orbitrap Mass Spectrometry

Proteins in the samples were identified and quantified using a high-resolution Hybrid Quadrupole–Orbitrap mass spectrometer (QExactive HF, Thermo Fisher Scientific, Waltham, MA, USA). Peptides were separated on a nano-HPLC Ultimate 3000 RSLC system (Dionex, Sunnyvale, CA, USA). Sample pre-concentration and desalting as well as separation of peptides were performed on a 25 cm Acclaim PepMap C18 column (75 µm inner diameter, 3 µm particle size, and 100 Å pore size). The gradient started with 4% B (80% acetonitrile (ACN) with 0.08% formic acid) and increased to 31% B in 60 min and to 44% B in an additional 5 min. It was followed by a washing step with 95% B. Mobile Phase A consisted of LC–MS grade H_2_O with 0.1% formic acid.

For mass spectrometric analysis applying data-dependent acquisition (DDA) the LC was directly coupled to a high-resolution Q Exactive HF Orbitrap mass spectrometer via a nano-electrospray ion source. MS full scans were performed in the ultrahigh-field Orbitrap mass analyzer in range of *m*/*z* 350−2000 with a resolution of 60,000. Maximum injection time was 50 ms and the automatic gain control (AGC) was set to 3e^6. The top 10 intense ions were subjected to Orbitrap for further fragmentation via high energy collision dissociation (HCD) activation over a mass range between *m*/*z* 200 and 2000 at a resolution of 15,000 with the intensity threshold at 4e^4. Ions with charge state +1, +7, +8, and >+8 were excluded. Normalized collision energy (NCE) was set at 28. For each scan, the AGC was set at 5e^4 and the maximum injection time was 50 ms. Dynamic exclusion of precursor ion masses over a time window of 30s was used to suppress repeated peak fragmentation. The LC–MS system was operated by Xcalibur 4.1.31.9 (Thermo Scientific, Waltham, MA, USA).

### 4.5. Data Processing for Protein Identification and Quantification

Acquired raw data was processed with the vendor specific platform for protein identification and quantification (Proteome Discoverer software, version 2.2.0.388, Thermo Fisher Scientific, San Jose, CA, USA). The database consisted of UniProt entries of *Mus musculus* (taxonomy id: 10090) as well as cRAP (common Repository of Adventitious Proteins: ftp://ftp.thegpm.org/fasta/cRAP/crap.fasta). Database search parameters applied were trypsin digestion, cysteine alkylation set to iodoacetamide. As dynamic modifications oxidation on methionine and N-terminal acetylation were allowed. Mass tolerance was 10 ppm, and fragment mass tolerance 0.02 Da. False discovery rate (FDR) performed using the integrated software tools was set to below 1% on peptide spectrum match (PSM) level and the minimal number of identified peptides per protein was set to 2.

For quantification DDA–Orbitrap data abundances of peptides were extracted from raw data using Proteome Discoverer. Raw abundances were normalized to the same peptide amount. Biological replicate abundances were calculated as the median of the technical replicate abundances. The study design of the statistical analysis was a non-nested approach based on pairwise ratios of the normalized, scaled abundances, comparing samples of the two treatments “Control” and “m193” and taking into account the four independent biological replicates per group. Peptide group ratios were calculated with the software Proteome Discoverer as the geometric median of all combinations of ratios from all the replicates (“m193”/”Control”). Protein ratios subsequently are the geometric median of the peptide group ratios. The *p*-values were adjusted with the Benjamini–Hochberg method. Protein level changes were considered statistically significant if the FDR-adjusted *p*-value was lower than 0.05, the fold change was at least two (fold change < −2 or > +2), and a minimum of one unique and at least two total peptides per protein.

### 4.6. RNA Data Analysis and Human Data

To extend the list of ECM-associated genes with genes not identified by MS-based analysis we manually compared genes at least 3-fold dysregulated in both miR-193a-driven FSGS and in glomeruli of human FSGS patients. ECM-association was determined by comparison to the matrisome resource database [15] and PubMed searches. More detailed data on the human samples were published before [18]. All FSGS samples were from patients with idiopathic FSGS. Controls were from “normal” regions of kidneys removed from Wilms’ tumor patients > 4 years of age (*n* = 3). All 4 FSGS patients were female, ranging in age from 17 to 29, and 2 were Caucasian and 2 African-American. The FSGS needle biopsy samples typically included 2–15 glomeruli. Urinary protein levels were 4.0, 5.4, 14.7, and 17.0 (g/day) and serum creatinine levels were 0.8, 1.1, 0.9, and 5.3 (mg/dL).

### 4.7. Histology and Antibody Stainings

Routine stainings (Periodic-Acid-Schiff, PAS; Acid Fuchsin Orange G AFOG) were performed on formalin-fixed paraffin-embedded histology samples after deparaffinization according to standard protocols. Immunohistochemistry were performed with the anti-collagen1 antibody (*ab34710*/Abcam, Cambridge, UK) and anti-fibrinogen antibody (*Dako A0080*/Agilent, Santa Clara, CA, USA) according to the manufacturer’s protocol.

### 4.8. Patents

There are no patents resulting from the work reported in this manuscript.

## Figures and Tables

**Table 1:** Significantly dysregulated genes in miR-193a-overexpressing glomeruli.

ID	Symbol	Gene Name	FC
P11680	CFP	Properidin	*
F8WJ05	ITIH1	Inter-alpha-trypsin inhibitor 1	*
O70570	PIGR	Polymeric Immunoglobulin Receptor	*
P50404	SFTPD	Pulmonary surfactant-associated protein D	*
Q566I6	C1R	Complement C1r	27.8
A8DUV3	HBA1	Hemoglobin subunit alpha 1	18.9
Q3UER8	FGG	Fibrinogen gamma chain	15.4
Q3TGR2	FGB	Fibrinogen beta chain	10.0
Q91X17	UMOD	Uromodulin	8.8
Q54AH9	HBB-B2	Hemoglobin subunit beta-2	8.7
Q542I3	CRP	C-reactive protein	7.6
A8DUK0	HBB-B1	Hemoglobin subunit beta-1	7.4
P01029	C4B	Complement C4-B	6.2
P07759	SERPINA3	Serine protease inhibitor A3K	6.1
A0A2P9DUN6	SERPINA1	Alpha-1-antitrypsin 1	5.6
Q00623	APOA1	Apolipoprotein A-I	5.3
P11087	COL1A1	Collagen alpha-1(I) chain	4.9
Q8VBX5	PPT1	Palmitoyl-protein thioesterase 1	4.8
Q9JMG7-2	HDGFL3	Hepatoma-derived growth factor-related protein 3	−5.2
O55186	CD59A	CD59A glycoprotein	−5.7

Differentially regulated genes were assessed by pairwise ratio comparison of glomeruli of miR-193a-overexpressing and wild-type mice; * not detectable in control samples; ID, protein accession number; FC, fold change miR-193a versus wild-type.

**Table 2:** ECM genes dysregulated in glomeruli of miR-193a-driven FSGS and FSGS patient samples.

Gene Name	Symbol	FC in Mouse ^1^	FC in Human ^2^	Description
Insulin Like Growth Factor Binding Protein 1	IGFBP1	100.6	9.1	IGF signaling stimulator
Angiotensin I Converting Enzyme 2	ACE2	95.7	5.9	vasoconstrictor, hypertension
Tubulointerstitial Nephritis Antigen	TINAG	65.4	4.3	glycoprotein
Defensin Beta 1	DEFB1	52.0	11.6	microbicidal, cytotoxic
Uromodulin	UMOD	44.2	51.3	calcium crystallization inhibitor, immune response
Phospholipase A1 Member A	PLA1A	42.2	5.0	phospholipase, signal transduction
Complement Factor I	CFI	37.9	6.9	complement cascade regulator
Serpin Family A Member 1f	SERPINA1	28.3	6.3	coagulation cascade
Osteopontin	SPP1	26.2	122.8	cell signaling modulator (inflammation, TGF-β, Wnt)
Kininogen 1	KNG1	26.1	26.3	coagulation cascade
EPH Receptor B2	EPHB2	20.8	3.2	ephrin signaling
Lumican	LUM	17.6	45.5	proteoglycan
Secreted Frizzled Related Protein 1	SFRP1	17.3	16.6	Wnt signaling modulator
Reelin	RELN	16.1	6.1	cell positioning/migration
WAP Four-Disulfide Core Domain 2	WFDC2	15.2	3.5	protease inhibitor
Complement Factor B	CFB	12.8	7.0	complement cascade regulator
Secretogranin V	SCG5	9.5	9.3	chaperone
Fibulin 1	FBLN1	8.4	3.1	glycoprotein
Mucin 1	MUC1	8.0	3.8	signal transduction
Complement C7	C7	7.5	5.3	complement cascade regulator
Semaphorin 4D	SEMA4D	6.9	5.0	glycoprotein, cell signaling
C-X-C Motif Chemokine Ligand 14	CXCL14	6.9	29.2	cytokine, immune response
Tissue Factor Pathway Inhibitor 2	TFPI2	6.9	3.5	coagulation cascade
Laminin Subunit Gamma 2	LAMC2	6.4	3.3	structural glycoprotein
Stanniocalcin 1	STC1	6.0	4.4	calcium/phosphate balance regulator
Glypican 4	GPC4	5.0	4.4	Glycoprotein
Clusterin	CLU	4.9	9.0	Chaperone
Serine Protease 23	PRSS23	4.8	25.7	serine protease
Insulin Like Growth Factor Binding Protein 3	IGFBP3	4.4	10.5	IGF signaling stimulator
Collagen Triple Helix Repeat Containing 1	CTHRC1	3.9	19.6	vascular remodeling, Wnt signaling
Asporin	ASPN	3.9	5.4	calcium-binding, TGF-β inhibitor
N-Acetylgalactosaminyltransferase 12	GALNT12	3.9	16.3	protein glycosylation
Sushi, Nidogen And EGF Like Domains 1	SNED1	0.27	0.33	insulin-responsive
Phospholipase A2 Receptor 1	PLA2R1	0.25	0.17	phospholipase signaling
Vascular Endothelial Growth Factor A	VEGFA	0.16	0.19	proliferation, migration, survival
Collagen Type IV Alpha 3 Chain	COL4A3	0.15	0.20	structural glycoprotein
Sclerostin	SOST	0.02	0.14	cell signaling (Wnt, TGF-β)

FC, linear fold change FSGS/control; ^1^ miR-193 model, published by Gebeshuber et al. [14]; ^2^ idiopathic FSGS patients, published by Bennett et al. [18].

## Supplementary Materials

Supplementary materials can be found at https://www.mdpi.com/1422-0067/21/6/2095/s1.

## Abbreviations

ACE2	angiotensin converting enzyme 2
AGC	automatic gain control
APOA1	Apolipoprotein A1
BMP	bone morphogenic protein
CFP	properdin
Col1a1	collagen 1
COL12A1	collagen type XII, alpha-1 chain
COL3A1	collagen type III, alpha-1 chain
CRP	C-reactive protein
C1R	complement C1r
C4B	complement C4-B
DDA	data-dependent acquisition
DTT	dithiotreitol, 1,4-bis-(sulfanil)-butan-2,3-diol
ECM	extra-cellular matrix
ESRD	end-stage renal disease
FGA, FGB, FGG	fibrinogen α, β and γ chains
FSGS	focal segmental glomerulosclerosis
GBM	glomerular basement membrane
HBA1	α-globin
HBB-B1	β1-globin
HBB-B2	β2-globin
HBSS	Hank’s Balanced Salt Solution
HCD	high energy collision dissociation
HDGFL3, HRP3	Hepatoma-Derived Growth Factor-Related Protein 3
HDL	high-density lipoprotein
HPLC-MS	high-performance liquid chromatography mass spectrometry
ITIH1	Inter-alpha-trypsin inhibitor heavy chain H1
IGF	insulin-like growth factor
IGFBP1	insulin growth factor binding protein 1
MS	mass spectrometry
NCE	normalized collision energy
NFκB	nuclear factor κB
PIGR	Polymeric immunoglobulin receptor
PPT1	Palmitoyl-protein thioesterase 1
RNA	ribonucleic acid
ROS	reactive oxygen species
TFA	trifluoro acetic acid
TGF-β	transformin growth factor beta
TLR4	Toll-like receptor 4
SERPINA1	serine proteinase A1
SERPINA3	serine proteinase A3
SFTPD	pulmonary surfactant-associated protein D
SOST	sclerostin
SPP1	osteopontin
TINAG	tubulointerstitial nephritis antigen
UMOD	Uromodulin, Tamm-Horsfall protein
VEGFA	vascular endothelial cell growth factor-alpha
WT1	Wilms’ tumor 1
